# Protocol Development for the Korean Survey for Cancer Survivorship and Preliminary Analysis of Employment Change’s Impact on Quality of Life and Psychological Health

**DOI:** 10.3390/cancers18020219

**Published:** 2026-01-09

**Authors:** Janine Marie Balbedina, Yeol Kim, Hye Joo Jang, Ha Yeong You, Jae Hyun Park, Hyun Woo Lee, Ji Soo Park, Yu Ri Choe, Kyu Won Jung

**Affiliations:** 1 Department of Public Health and AI, Graduate School of Cancer Science and Policy, National Cancer Center, Goyang 10408, Republic of Korea; jarieblbdna@gmail.com; 2 National Cancer Survivorship Center, National Cancer Center, Goyang 10408, Republic of Korea; hyejoo1121@ncc.re.kr (H.J.J.); uh0@ncc.re.kr (H.Y.Y.); 3 Department of Social and Preventive Medicine, School of Medicine, Sungkyunkwan University, Suwon 16419, Republic of Korea; pjaehyun@skku.edu; 4 Department of Hematology-Oncology, School of Medicine, Ajou University, Suwon 16419, Republic of Korea; hwlee71@gmail.com; 5 Cancer Prevention Center, Yonsei Cancer Hospital, Seoul 03722, Republic of Korea; pmjisu@yuhs.ac; 6 Department of Family Medicine, Hwasun Hospital, Chonnam National University, Hwasun 58128, Republic of Korea; hiyuriya@cnuh.com; 7 Division of Cancer Registration and Surveillance, National Cancer Control Institute, National Cancer Center, Goyang 10408, Republic of Korea; ara@ncc.re.kr

**Keywords:** cancer survivors, depression, anxiety, quality of life, employment

## Abstract

**Background/Objectives**: The Korean Survey for Cancer Survivorship (KSCS) aims to comprehensively assess cancer survivors’ health behaviors, quality of life (QoL), and socioeconomic challenges. This study evaluated the feasibility of the KSCS protocol and identified key factors influencing psychological health and QoL among cancer survivors. **Methods**: The nationwide survey targeted survivors diagnosed with breast, colorectal, liver, lung, stomach, prostate, and gynecological cancers who had completed active treatment within 1 to 10 years. The respondents were given the option to participate in the survey either online or in-person. The questionnaire has 229 questions, including internationally validated tools such as the EQ-5D-3L, PHQ-9, and GAD-7. **Results**: A total of 983 cancer survivors completed the survey (92.7% online, 8.3% in-person) and were categorized by post-diagnosis duration. Survivors diagnosed within 1–3 years reported higher rates of moderate-severe depression (11.4% vs. 8.3%), moderate-severe anxiety (5.9% vs. 5.1%), and poorest QoL (63.0% vs. 50.9%) compared to those diagnosed more than 5 years ago. Employment changes, such as loss of job, change of workplace, or work leave, were significantly associated with worse health outcomes, including higher rates of moderate-severe depression (OR = 4.39; 95% CI 2.43–7.96), moderate-severe anxiety (OR = 3.63; 95% CI 1.68–0.88), and having extreme QoL problems (OR = 6.37; 95% CI 2.03–20.00). **Conclusions**: The KSCS protocol is feasible for nationwide implementation and provides comprehensive data on health, psychological, and socioeconomic challenges among cancer survivors. Preliminary findings highlight employment’s critical role in cancer survivors’ well-being and the need for survivorship care that integrates socioeconomic and clinical factors.

## Simple Summary

The Korean Survey for Cancer Survivorship (KSCS) is a nationwide multi-center study that aimed to systematically investigate cancer survivors’ problems after cancer treatment. This study reports the protocol of KSCS and the process of its development. The study aimed to evaluate the feasibility of the KSCS protocol and explore the preliminary associations between employment change and cancer survivors’ well-being. KSCS targets about 5000 cancer survivors diagnosed with breast, colorectal, liver, lung, stomach, prostate, and gynecological cancers who had completed active treatment within 1 to 10 years. Preliminary analysis showed that employment change is strongly associated with worse psychological health and extreme quality of life (QoL) problems. The study demonstrated that the KSCS protocol is feasible for nationwide application and that employment-related factors play a significant and independent role in shaping cancer survivors’ mental health and overall QoL. The KSCS provides valuable evidence for comprehensive cancer survivorship care and policy development.

## 1. Introduction

Cancer survivorship has emerged as a crucial area of focus within public health, driven by advances in early detection and treatment that have led to improved survival rates [1] for many types of cancer. As a result, the number of individuals living after cancer diagnosis has grown. The latest data from the International Agency for Research on Cancer in 2022 estimated that the 5-year all-cancer prevalence worldwide was 53.5 million [2]. In Korea, recent data from the Korea Central Cancer Registry show that prevalent cases in 2021 reached over 2.4 million, with a 5-year relative survival rate of 72.1% between 2017 and 2021 [3]. The increasing cancer survival rate highlights the importance of understanding the unique challenges cancer survivors face.

Cancer survivors often experience a range of long-term effects, including physical, psychological, and socioeconomic difficulties that can impact their quality of life (QoL), as well as issues with returning to work or maintaining previous levels of social engagement [4,5,6]. However, a recent study highlighted that there is still lack of information and support among patients living with and beyond cancer [7].

Understanding the health behaviors, mental well-being, and QoL of cancer survivors is vital for developing effective survivorship care programs. Additionally, cancer diagnosis has major implications for employment and financial outcomes at both the individual and family levels. Comprehensive data collection can help develop fitted and evidence-based healthcare policies, ensuring that cancer care extends after treatment to cover all the aspects of survivorship needs.

Several developed countries have already been conducting nationwide surveys on cancer survivorship. In the United States, the National Coalition for Cancer Survivorship has conducted the annual State of Survivorship Survey since 2019, documenting persistent financial, quality-of-life, and employment-related challenges among cancer survivors following diagnosis and treatment [8]. Similarly, Canada’s Transition Study, a large population-based survey conducted across multiple provinces, examined survivors within the first few years after completing primary treatment and reported substantial ongoing physical, emotional, and practical difficulties [9]. These national surveys highlight the importance of large-scale, systematic data collection in identifying unmet survivorship needs and guiding cancer survivorship policy and care planning.

Looking into South Korea’s cancer survivorship efforts, several studies have already explored the experiences of cancer survivors. A 2013 study using data from the Korea National Health and Nutrition Examination Survey (KNHANES) reported that 783 adult cancer survivors (mean age: 60.9 years) had significantly poorer quality of life compared with 36,456 noncancer controls, with pain and discomfort being the most frequently reported issue [10]. Survivors also reported greater difficulties with mobility, performing daily activities, pain/discomfort, and anxiety/depression compared to controls [10]. Building on these findings, it is important to further explore how specific symptoms and economic factors impact the quality of life of cancer survivors. A 2017 study which assessed the quality of life of 145 Korean gastrointestinal cancer survivors found that high symptom burden, characterized by persistent pain, fatigue, and other debilitating symptoms, as well as economic difficulties, such as high medical costs and financial hardship, were significantly associated with poorer quality of life [11], highlighting the need for comprehensive strategies focused on managing these symptoms and providing financial support to improve survivors’ overall well-being. These results are further supported by a retrospective study that utilized the 6th and 7th (2014, 2016, 2018) KNHANES results, which analyzed 205 employed survivors and found that age, sex, depression, and subjective health status significantly affected quality of life [12].

Although these studies contribute to the knowledge of cancer survivorship in Korea, they have several limitations including recruitment of participants from only a few hospitals [12] and are considered as cancer-specific or small group surveys with having a small number of cancer survivors included in the analysis [10,11,12] that are deemed insufficient in coming up with national level cancer care programs and policies. This underscores the need for a nationwide survey for cancer survivorship.

The Korean Survey for Cancer Survivorship (KSCS) was conducted to address knowledge gaps on cancer survivorship in Korea by providing comprehensive data on cancer survivors’ health behaviors, quality of life, and socioeconomic challenges. There is a need to analyze sociodemographic and clinical characteristics because these factors can have varying short-term and long-term physical, psychological, and social impacts on survivors. Understanding these variations is critical for developing targeted, effective interventions. A large-scale nationwide survey for cancer survivorship is necessary to identify gaps in cancer care and develop evidence-based initiatives to improve the quality of life, health outcomes, and support systems for cancer survivors at the national level. Findings from the KSCS are expected to play a crucial role in guiding healthcare practices and policy decisions aimed at enhancing cancer survivorship support.

## 2. Materials and Methods

### 2.1. Development of the Questionnaire for the Korean Survey for Cancer Survivorship

A multidisciplinary, expert committee developed the protocol for the KSCS. The survey questionnaire was constructed based on the Korea Health Panel Survey and the KNHANES with the aim of comparing the health status and health behaviors of the cancer survivors and the Korean general population. Some of the questions were revised to better align with the specific experiences of cancer survivors. The survey questionnaire was approved by the expert committee.

The survey questionnaire is composed of 229 primary questions and 82 sub-questions divided into seven parts. Part 1 asks about cancer survivors’ cancer experience, including cancer discovery and diagnosis, cancer treatment/s undergone, cancer recurrence, caregivers, and hospitalization experience and costs. Part 2 is about comorbidity management, which asks about presence or absence of chronic diseases before cancer diagnosis and how it was managed. Part 3 is about cancer survivors’ quality of life, which is further subdivided into sub-sections: general quality of life, quality of life of cancer patients, sex life, and mental health. Internationally validated tools such as the EORTC Core Quality of Life questionnaire (EORTC QLQ-C30), EQ-5D-3L (European Quality of Life 5 Dimensions 3 Level Version), PHQ-9 Patient Depression Questionnaire, and General Anxiety Disorder-7 (GAD-7) Questionnaire were utilized and included in this part. Licenses to use these tools were secured accordingly. Responses on the EQ-5D-3L were grouped according to the presence of problems in each of the dimensions. Participants who responded with option 1 in all five categories were classified under the “No problems at all” group. However, if the participants answered option 2 in any of the dimensions, they will be classified under the “With some problems” group. Similarly, when the participants chose option 3 in any of the dimensions, they were classified under the “With severe problems” group. Responses on the PHQ-9 and GAD-7 were grouped according to the official severity categories of each tool. For the PHQ-9, participants were classified into the following groups based on their total scores: 0–4 as “Minimal or no depression,” 5–9 as “Mild depression,” 10–14 as “Moderate depression,” 15–19 as “Moderately severe depression,” and 20–27 as “Severe depression.” For the GAD-7, participants were classified into the following groups: 0–4 as “Minimal or no anxiety,” 5–9 as “Mild anxiety,” 10–14 as “Moderate anxiety,” and 15–21 as “Severe anxiety.” For analysis, we merged the moderate and severe categories for both the PHQ-9 and GAD-7. This was done to address the relatively small number of participants in the severe category, which could limit statistical power and stability of the estimates.

Part 4 focuses on the unmet needs of cancer survivors related to their condition in terms of information and education, psychological problems, physical symptoms, hospital facilities and services, family/interpersonal issues, religious/spiritual problems, and social support. Part 5 is about policy-related indicators, which are further divided into 5 sub-sections: financial status and burden of medical expenses, changes in economic activity and employment, cancer screening, vaccination, and complementary and alternative therapies. Part 6 asks about the health behaviors of cancer survivors and includes questions on weight management, diet intake and changes, alcohol drinking, smoking, physical activity, and sleep patterns. Finally, part 7 asks about cancer survivors’ demographic characteristics with questions about gender, date of birth, marital status, educational level, area of residence, household living conditions, income, and health insurance.

Employment change, the main predictor variable examined in the preliminary analysis of this paper, was measured as a binary variable using the question, “Has there been any change in your work or employment after your cancer diagnosis?” Those who answered “Yes” were asked to answer a follow-up question, “What change, if any, has occurred since your cancer diagnosis?”, with choices including “on paid leave of absence, on unpaid leave of absence, loss of job, change of workplace, started own business, and others.”

### 2.2. Recruitment Process

**Figure 1:**
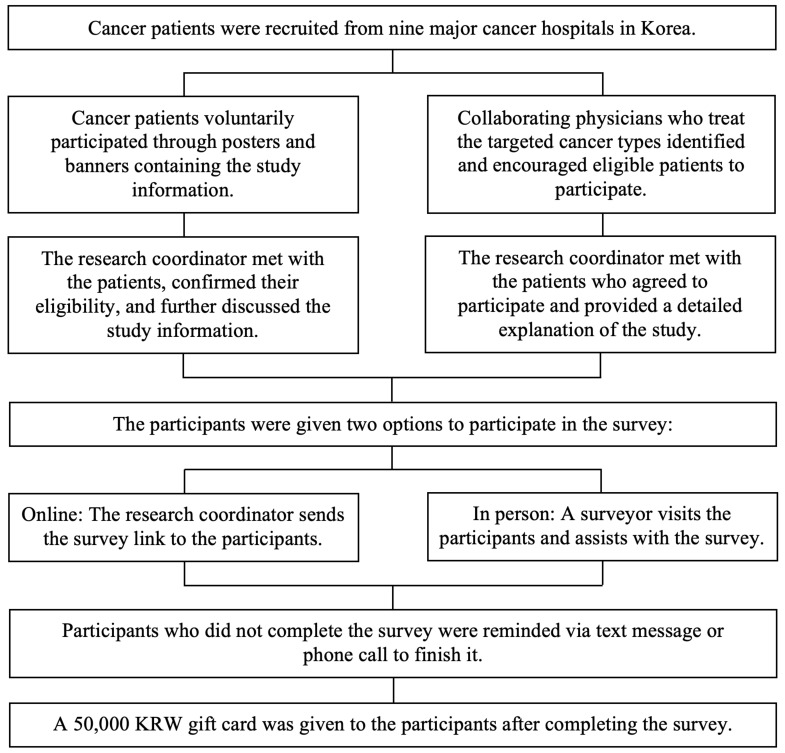
Summary of the participant recruitment process of the Korean Survey for Cancer Survivorship.

The study participants were recruited from nine major cancer hospitals across Korea. Recruitment was done in two ways: (1) voluntary participation through posted study information in hospitals, or (2) direct recruitment by the physicians and research coordinator. Figure 1 provides a summary of the participant recruitment procedure through these two approaches.

**Figure 2:**
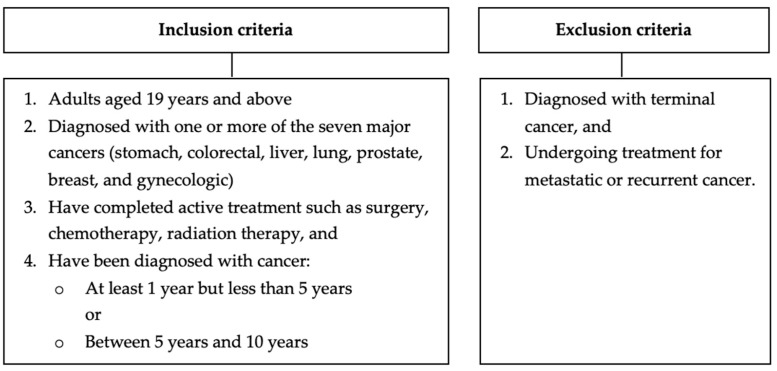
Eligibility criteria for the target survey participants of the Korean Survey for Cancer Survivorship.

Through the voluntary participation approach, the study was promoted in hospitals through posters and banners. The promotional materials were placed in areas where cancer survivors can easily see them, such as the hallways, admissions office, and billing office. The study goals, participants’ eligibility criteria, and survey procedures were included in the materials. The research coordinator’s phone number and a QR code linked to the application form were also included. Interested patients applied to participate either by completing the application form through the QR code or by directly contacting the research coordinator by phone call. Once the participant’s application was received through the QR code, the research coordinator promptly contacted the patient to arrange a time and location to meet at the hospital. Some patients directly called the research coordinator, who then briefly discussed the study via phone call and arranged a meeting with them. If an immediate meeting was not possible, an appointment was scheduled instead. During the meeting, the research coordinator reconfirmed the patients’ study eligibility and provided further information about the study. Figure 2 shows the eligibility criteria for the target survey participants.

Through the direct recruitment approach, collaborating physicians who treat the targeted cancer types identified and encouraged eligible patients to participate in the study during their routine outpatient clinical consultations. After the patients agreed to participate in the study and finished their consultation, the research coordinator met with them and provided a detailed explanation of the study.

Informed consent was obtained from all participants, and a small gift was given upon confirmation of participation. Participants were given two options for survey participation: (1) online, with the research coordinator sending them the survey link to answer independently, or (2) in person, with a surveyor visiting them and assisting with the survey. Those who did not complete the survey right away were sent reminder text messages to encourage them to complete the survey. Upon survey completion, a 50,000-KRW (Korean won) gift card was given to the participants.

### 2.3. Eligibility Criteria for Participants

The inclusion criteria for the cancer survivorship survey are as follows: (1) adults aged 19 years and above, (2) diagnosed with one or more of the seven major cancers in Korea (stomach, colorectal, liver, lung, prostate, breast, and gynecologic), (3) completed active treatment such as surgery, chemotherapy, radiation therapy, and (4) diagnosed with cancer for at least one year. Excluded from the study are anyone: (1) diagnosed with terminal cancer and/or (2) undergoing treatment for metastatic or recurrent cancer.

### 2.4. Number of Participants

The sample size goal of this study was 5000 cancer survivors, comprising 4000 diagnosed for at least one year to less than five years, and 1000 long-term survivors diagnosed within five to ten years. The target number of participants was determined based on the seven major cancer types in Korea, namely lung, stomach, colorectal, breast, prostate, liver, and gynecologic cancers. They were identified by their primary cancer diagnosis corresponding to one of these seven cancer types. Gynecologic cancers, including uterine and ovarian cancers, were grouped together in this study.

For the 1–5-year post-diagnosis group, the target numbers were 700 for lung cancer; 600 each for stomach, colorectal, and breast cancers; and 500 each for liver, prostate, and gynecologic cancers. For the long-term group (5–10 years post-diagnosis), approximately 150 survivors were targeted for each cancer type. These target sample sizes were determined based on both the prevalence and the survival rates of each cancer type in Korea for the two-year follow-up survey (see Table A1). From the 1–5-year group, an estimated 2800 survivors are expected to remain for follow-up after two years. Taking into account the response rate, the KSCS aims to reenroll 2000 survivors for the follow-up survey. Follow-up surveys will be conducted every two years thereafter (see Table A2).

The study participants were then grouped by period after cancer diagnosis (<3 years, 3–5 years, and >5 years) for analysis. Survivors in the <3 years and 3–5 years groups will be followed up after two years to assess the proportion who show recovery at follow-up.

### 2.5. Statistical Analysis

The association between demographic characteristics and period post-diagnosis was evaluated using chi-square test. The association of clinical factors, including period after diagnosis and cancer type, socio-demographic factors, including sex, age, monthly income, and current employment status, and health behavioral factors, including smoking, drinking, and physical activity to health outcomes, including depression and anxiety levels, and quality of life was evaluated using ordinal logistic regression analysis. Univariate analyses were first conducted to examine crude associations between changes in employment and health outcomes. Multivariable analyses were then performed to estimate adjusted associations while accounting for relevant sociodemographic and clinical factors; therefore, only multivariable-adjusted results are presented to provide more interpretable estimates. All statistical analyses were performed using STATA software ver. 14 (Stata Corp. L.P., College Station, TX, USA).

## 3. Results

### 3.1. Protocol Implementation

The implementation of the study protocol demonstrated both its strengths and areas for improvement. The use of both online (92.7%) and in-person (8.3%) survey methods provided flexibility and practicality, adjusting to participants’ preferences and enhancing accessibility. The data collection yielded high-quality and complete responses, indicating that the survey questionnaire and design were clear and easy to understand. These findings assert the feasibility of the protocol while also emphasizing the need for refinement to optimize efficiency and adaptability in the subsequent nationwide study.

### 3.2. Survey Outcomes

A total of 983 participants in the survey were categorized by period post-diagnosis. Of these, 400 (40.7%) were diagnosed less than 3 years ago, 249 (25.3%) were diagnosed within 3–5 years, and 334 (34.0%) were diagnosed more than 5 years ago. The sex distribution showed that females comprised the majority (64.0%), and most of the participants were aged 50–65 years old (49.6%). The majority of participants hold a university degree (48.4%) and reported monthly incomes between 2,000,000 and 4,000,000 KRW (38.0%). Current employment status analysis indicated that 32.0% were employed, but 49.7% were unemployed or not actively seeking employment. Breast cancer (33.5%) was the most common type of cancer diagnosis. Additional sociodemographic and clinical characteristics of the participants are presented in Table 1. Data on the health behaviors of the participants showed that 75.5% of participants were non-smokers and only 5.5% were current smokers. Drinking status showed the highest percentage for drinkers who drink less than 2 times per week (33.1%), and the majority of the participants do physical activity such as walking for 5–7 days per week (65.7%). Among all these factors, gender (*p* = 0.001), age (*p* = 0.000), cancer type (*p* = 0.001), and drinking status (*p* = 0.003) showed significant association with the period post-diagnosis.

The associations between clinical and socio-demographic factors with health outcomes among cancer survivors are presented in Table 2. Ordinal logistic regression was used for outcomes measured on ordered categorical scales. Although some outcome categories were unevenly distributed, this approach was selected to preserve the ordinal structure of the outcome variables and avoid information loss associated with dichotomization. The model estimates the association between predictor variables and the likelihood of being in a worse outcome category, using all levels of the ordered scale simultaneously. Regarding period post-diagnosis, anxiety and depression did not significantly differ across groups, but quality of life was significantly lower among survivors diagnosed within the past three years compared with those diagnosed more than five years ago (odds ratio (OR) = 1.55, 95% confidence interval (CI) = 1.16–2.08). In terms of socio-demographic factors, female cancer survivors showed increased likelihood of anxiety (OR = 1.59, 95% CI = 1.15–2.19), depression (OR = 1.88, 95% CI = 1.43–2.48), and poor quality of life (OR = 2.72, 95% CI = 2.09–3.55) than males. This finding is consistent with the cancer type analysis, where breast cancer survivors also showed significantly poorer outcomes across all three measures. Younger survivors, particularly those under 50 years, were more likely to experience anxiety (OR = 3.84, 95% CI = 2.50–5.92), depression (OR = 3.84, 95% CI = 2.62–5.64), and poor quality of life (OR = 3.02, 95% CI = 2.09–4.37) compared to those aged over 65. Survivors with a monthly household income below 2,000,000 KRW also had increased likelihood of anxiety (OR = 2.05, 95% CI = 1.32–3.20), depression (OR = 2.06, 95% CI = 1.40–3.02), and poor quality of life (OR = 1.50, 95% CI = 1.02–2.20) compared to those with income above 4,000,000 KRW. In addition, being unemployed was associated with greater likelihood of depression (OR = 1.38, 95% CI = 1.03–1.84) and poor quality of life (OR = 1.91, 95% CI = 1.43–2.54) compared with being employed.

Given the significant findings related to economic factors, we further analyzed the association of employment changes on the health outcomes of cancer survivors through univariate and multivariate models. The results are presented in Table 3. Multivariable odds ratios were adjusted for the effects of age, gender, education level, income level, cancer type, and cancer stage. The results indicated significant associations between changes in employment and depression level, anxiety level, and quality of life. Specif-ically, cancer survivors who experienced a change in employment were more likely to report moderate-severe anxiety (OR = 3.34; 95% CI = 1.51–7.41), moderate-severe de-pression (OR = 4.18; 95% CI = 2.28–7.69), and poorer quality of life (OR = 5.73; 95% CI = 1.76–18.66) compared to those who did not experience a change in employment. All associations remained statistically significant in both univariate and multivariate analyses, highlighting the impact of change in employment on cancer survivors’ quality of life and psychological health.

**Figure 3:**
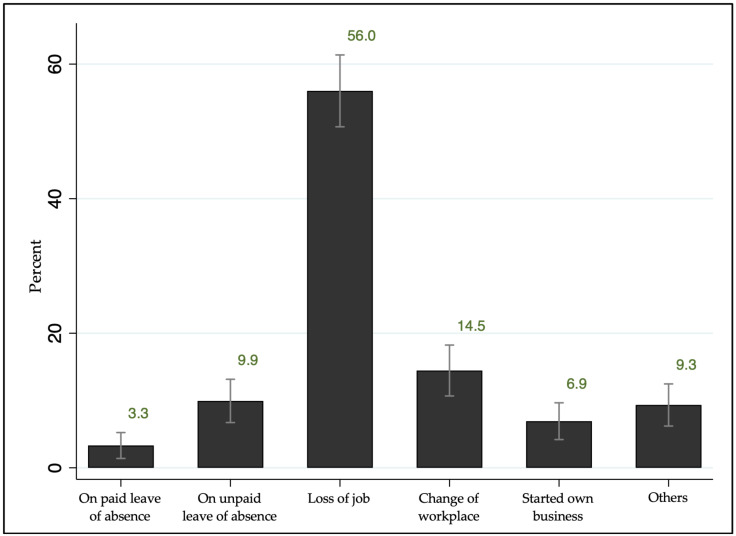
Proportion of cancer survivors with changes in employment status (n = 332) after cancer diagnosis from the Korean Survey for Cancer Survivorship, with error bars indicating 95% confidence intervals, illustrating the uncertainty around each estimate. Loss of job shows a high prevalence with a relatively narrow confidence interval, indicating a stable estimate, whereas wider confidence intervals for other categories suggest lower precision due to smaller sample sizes.

## 4. Discussion

This study initiated the nationwide survey for cancer survivorship in Korea. Conducting such a survey is critical for understanding the multifaceted challenges that cancer survivors face and addressing their diverse needs. As advancements in cancer detection and treatment have led to increased survival rates, it has become critical to shift from merely extending cancer patients’ lives to improving long-term health outcomes of survivors [13]. This nationwide survey allows for a systematic collection of data on various aspects of cancer survivorship, such as physical, psychological, social, and financial concerns, while capturing differences across demographic and geographic groups. This comprehensive approach ensures that the findings are representative and can inform evidence-based policies and interventions tailored to the unique needs of cancer survivors. In Korea, where the population of survivors is growing rapidly [3], this survey is a crucial step toward identifying gaps in cancer care, addressing inequities, reducing disparities, and improving survivorship care plans to better support individuals throughout their cancer experience.

Furthermore, this study initiated the development of a protocol for the implementation of a nationwide survey for cancer survivorship in Korea. The protocol was approved by a multidisciplinary expert committee, ensuring that it incorporates a wide range of perspectives and expertise. The protocol introduced a systematic way of obtaining participants wherein the survey can be completed either in-person or by online access, and strict compliance with the inclusion criteria is followed and closely monitored. Furthermore, the survey is implemented in multiple centers across the country, enhancing generalizability, diversity in perspectives, increased sample size, and efficiency in data collection. The survey also includes diverse questions covering various aspects of a cancer survivor’s life. This nationwide protocol can serve as a foundation for long-term monitoring, enabling the evaluation of changes over time and the effectiveness of survivorship care policies.

The Transition Study survey implemented in Canada provides valuable insights into cancer survivors’ experiences during their first three years post-treatment, particularly focusing on the transition from specialized cancer care to general healthcare systems [9]. Similarly, the State of Survivorship Survey in the United States explored a broader timeframe, offering important perspectives on post-treatment care and the financial implications of cancer on survivors [8]. Complementing these studies, our survey included the period after diagnosis, emphasizing the significance of understanding health outcomes such as quality of life, depression, and anxiety levels at various stages of survivorship. In addition to health outcomes and quality of life, economic impacts of cancer diagnosis and treatment are also crucial information, particularly how the cancer survivors’ work abilities are affected [14,15,16,17]. Several studies have shown that work is a crucial aspect of a person’s realization of self-worth and in their overall quality of life [18,19,20]. This study’s survey questionnaire also looked into changes in employment status of cancer survivors before and after cancer diagnosis, which gave relevant information on how cancer affects a person’s day-to-day life dynamics.

In Korea, a survey among 949 lung cancer survivors was conducted by the Samsung Medical Center in 2020 using the Korean version of the self-reported Cancer Survivors’ Unmet Needs (CaSUN-K) scale [21]. The CaSUN-K was found to be a reliable and valid tool for assessing the unmet needs of cancer survivors; however, due to its limited scope, it primarily focuses on specific categories of needs and may not comprehensively capture the evolving and diverse challenges faced by cancer survivors across different stages of their journey [21]. Expanding the scope of such tools can further enhance our understanding and guide the development of holistic survivorship care plans. The nationwide protocol developed in this study addresses this need by incorporating a comprehensive framework that evaluates diverse aspects of cancer survivorship, ensuring the inclusion of varied survivor experiences and needs.

Due to advances in cancer treatments, cancer survival of five or more years following a cancer diagnosis is expected to continuously increase [1]. These improvements in survival rates underscore the importance of assessing cancer patients’ health outcomes across different stages of survivorship. This study provided evidence that quality of life differs significantly across periods of post-diagnosis, wherein survivors in the early survivorship stage (<3 years after diagnosis) experience poorer quality of life compared to those in long-term survivorship (>5 years after diagnosis). A study that analyzed the KNHANES 2008–2012 data involving 1020 cancer survivors found that time since diagnosis significantly influenced health-related quality of life, particularly mobility, which may be explained by cancer treatment and relapse commonly occurring within 3–5 years [22]. The American Cancer Society also reported in their Cancer Treatment & Survivorship Facts & Figures for 2014–2015 that cancer-related pain may be present at diagnosis and is associated with poorer quality of life, depression, and functional limitations [23]. However, our study did not find any significant association between depression and anxiety levels and periods of post-diagnosis. This finding is in contrast with the results of previous studies wherein psychological health was found to be poorer in recently diagnosed cancer survivors [7,24]. A 5-year observational cohort study that looked into early breast cancer patients found that almost 50% of the participants reported depression, anxiety, or both within 1 year after diagnosis, but fell to only 15% in the fifth-year post-diagnosis [24]. A study in England that looked into suicide risk following cancer diagnosis among 4.8 million patients reported a 0.08% suicide mortality during follow-up, with the first six months post-diagnosis identified as a particularly critical period [7]. The contrasting findings in our study could be attributed to differences in study design, population characteristics, or cultural factors that influence psychological resilience and coping mechanisms. Unlike previous studies, our survey included participants from diverse cancer types and survivorship stages, which may have diluted specific trends observed in more narrowly focused cohorts. In addition, the cross-sectional design of the study limits the ability to capture temporal changes in psychological health across survivorship stages. Potential selection bias may also have influenced the findings, as survivors experiencing more severe anxiety or depression may have been less likely to participate, particularly given the high proportion of online survey responses. These methodological considerations may partly explain the absence of significant differences in anxiety and depression across periods post-diagnosis observed in this study. While our study did not find significant associations between psychological health and post-diagnosis periods, ongoing monitoring of mental health outcomes in diverse survivor populations can help identify subtle trends and emerging needs. These data will be crucial for refining psycho-oncology services and tailoring interventions to specific survivor groups.

To the best of our knowledge, this is the first study to assess the impact of employment changes on the quality of life and psychological health of cancer survivors. Findings from this study revealed that cancer survivors who experienced changes in employment were more likely to report poorer psychological health and have reduced quality of life. Specifically, they had a higher likelihood of experiencing moderate-to-severe anxiety (OR = 3.34) and moderate-to-severe depression (OR = 4.18), with odds ratios suggesting they were approximately four times more likely to report these mental health issues. Additionally, they were more likely to report poorer quality of life, with an OR = 5.73, indicating they were approximately six times more likely to have a lower quality of life. These associations remained consistent in both univariate and multivariate analyses, highlighting the critical impact of employment transitions on mental health and well-being of cancer survivors. Employment provides a sense of fulfillment, stability, and self-worth for most people. Although the present study was not designed to formally test a specific theoretical model, the observed associations can be interpreted within established frameworks linking work, stress, and health. In particular, the Stress Process Framework suggests that employment plays a central role in shaping psychological well-being through multiple pathways, including financial security, social identity, daily structure, and perceived self-worth [25]. Moreover, the Latent Deprivation Model proposes that employment provides essential psychosocial benefits beyond income, including social identity, daily structure, and a sense of purpose, and the loss of which may adversely affect mental health [26]. For cancer survivors, maintaining or resuming employment can help restore a sense of stability and autonomy during this difficult period [18]. However, changes in employment, including cancer-related job loss and retirement, have been associated with psychosocial distress in both the short and long term. A study that looked into employment outcomes among cancer survivors confirmed that they are more likely to experience unemployment or early retirement and less likely to resume work, with 26% to 53% losing their jobs or stopping work within 72 months of diagnosis [27]. Furthermore, compared to the normal population, there is a higher percentage of temporary adjustments in schedules, hours, wages, and reduced work ability among cancer survivors [27]. For adults of working age, cancer-related work impairment represents a major burden [28]. Although some survivors opt for early retirement or reconsider their career plans due to illness, many experience the undesirable effects of cancer on employment, with significant consequences on the psychological and financial well-being of both survivors and their families [18,29]. Financial toxicity from reduced or loss of income and increased costs following a cancer diagnosis is associated with heightened distress, poorer quality of life, and poorer treatment adherence [15,16]. While these studies primarily looked into the effect of cancer on the work ability and situation after diagnosis and treatment, they did not analyze the direct association of changes in employment with the health outcomes of cancer survivors. The survey questionnaire developed by our study looked into these associations, revealing significant findings that are highly notable information to more deeply understand how the economic and health aspects affect each other, particularly among cancer survivors.

Studies that further compared factors influencing return to work among salaried and self-employed cancer survivors found that the self-employed group reported significantly greater reductions in work hours, more financial and occupational challenges related to cancer, and lower overall health, quality of life, and ability to work [17,18]. These findings highlight the need for further research into how cancer affects various aspects of employment, including job status, working hours, job retention, and productivity, to better capture the full implications of the illness on individuals’ occupational experiences.

Given the cross-sectional design of the study, these findings should be interpreted with caution, particularly regarding the observed association between employment change and poorer health outcomes. Although this study’s findings showed that employment change was strongly associated with poorer psychological health and quality of life, the relationship between employment status and these outcomes is likely bidirectional. A systematic review reported that mental health problems are associated with lower employment retention and function as both a consequence and a risk factor for poor employment status [30]. Similarly, longitudinal evidence has shown that the development of mental health problems was associated with a significantly increased probability of transitioning to non-employment [31]. While prior studies suggest a bi-directional relationship, the cross-sectional design of the present study means that reverse causality cannot be excluded. Nevertheless, regardless of directionality, the findings underscore employment change as an important indicator of vulnerability among cancer survivors, highlighting the need for early identification and support for survivors experiencing employment instability. Planned longitudinal follow-up in future phases of the Korean Survey for Cancer Survivorship will allow temporal assessment of these relationships and provide clearer insight into causal pathways.

### Strengths and Limitations

This study has some limitations. First, as the survey was in its early period of implementation, the sample size used in the current analysis was relatively small. Second, the cross-sectional design of the study limits causal inferences between variables such as change in employment and health outcomes. Third, self-reported data were utilized in the study, which may be subject to recall bias and social desirability bias. Fourth, although the survey collected a wide range of variables relevant to cancer survivorship, not all potentially influential factors were included in the present analysis due to the scope and focus of this study. Fifth, certain contextual and experiential information on cancer survivors’ experiences that may affect the interpretation of the findings was not collected through the initial questionnaire. Lastly, the survey targeted cancer survivors receiving follow-up care at hospitals, which may have introduced selection bias by underrepresenting socially and economically vulnerable populations and limiting the generalizability of the findings, particularly given the observed association between lower income and poorer psychological and quality-of-life outcomes.

Despite its limitations, this study has several notable strengths. KSCS is a large-scale, multi-center survey that represents one of the initial efforts to systematically assess cancer survivorship in Korea, utilizing nationwide data that are tailored to the local cultural and healthcare context. This broad coverage enhances the relevance of the findings and provides valuable insights into the lived experiences and needs of Korean cancer survivors. Furthermore, the study’s design establishes a strong foundation for refining research methodologies and developing more comprehensive survey tools for future large-scale studies. Moreover, its findings contribute evidence that highlights critical areas for intervention, such as psychological health, quality of life, and employment challenges among cancer survivors. These findings offer practical directions for policymakers, healthcare providers, and support organizations working to improve cancer survivorship care in Korea.

This study is based on pilot data (approximately 1000 participants) from the nationwide protocol. Once the target recruitment of 5000 cancer survivors is completed, the survey will be able to provide more representative and stable estimates at the national level. Building on the current study findings, the next phase of this research will focus on longitudinal follow-up of cancer survivors to monitor changes in their overall health status, psychological well-being, quality of life, and economic situation over time. These longitudinal data will allow identification of predictors of recovery and vulnerability, particularly among socioeconomically disadvantaged and working-age survivors, to support the development of timely, targeted, and personalized survivorship support strategies. The questionnaire will be further refined and expanded to capture more detailed contextual information on cancer survivorship experiences, which will help improve understanding of survivorship patterns and their effects on psychological health, quality of life, and socioeconomic outcomes. In addition, future analyses will explore disparities across demographic, health behavior-related, clinical, and employment-related subgroups to further develop equity-oriented survivorship policies and care models. Further efforts will be made to enhance data collection by incorporating diverse survivor populations, including those outside hospital settings, to further improve representativeness and depth of understanding of cancer survivors’ experiences and needs. These future directions will strengthen the role of the KSCS as a sustainable national evidence platform to guide survivorship care planning, policy development, and long-term support for cancer survivors in Korea.

## 5. Conclusions

This study highlights the feasibility of the Korean Survey for Cancer Survivorship (KSCS) protocol and demonstrates its capacity to capture comprehensive, high-quality data reflecting the multidimensional experiences of cancer survivors nationwide. The mixed online and in-person survey implementation produced high-quality data and effectively captured the diverse experiences of survivors across cancer types and stages of survivorship. Preliminary findings revealed significant differences in health outcomes across sociodemographic and clinical groups, with women, younger survivors, and those with lower income showing greater risk of anxiety, depression, and poorer quality of life. These findings suggest that survivorship care should prioritize early identification and targeted support for vulnerable subgroups to prevent persistent psychological distress and deterioration in quality of life. In particular, employment change emerged as a strong, independent determinant of poorer health outcomes, underscoring the need to address the socioeconomic challenges that cancer patients experience after diagnosis and treatment. Assessing employment change during survivorship follow-up may serve as a practical screening indicator to identify survivors at increased risk of adverse mental health and quality-of-life outcomes and facilitate timely referral to psychosocial and supportive services. These findings highlight the importance of integrating psychosocial and economic considerations into cancer survivorship care planning in Korea.

Despite the inherent limitations of the pilot dataset, this study establishes a critical foundation for the full nationwide rollout of the protocol and future longitudinal assessments. As the survey plans to expand to include the target sample of 5000 cancer survivors, it will enable more representative national estimates and facilitate monitoring of changes in health and socioeconomic outcomes over time among cancer survivors. The KSCS will serve as a key evidence-generating platform to inform tailored interventions, guide equitable cancer survivorship policies, and strengthen long-term capacity to support cancer survivors across clinical, psychological, and socioeconomic domains.

## Figures and Tables

**Table 1:** General characteristics of the participants in the Korean Survey for Cancer Survivorship.

Characteristics	Total N (%) 983 (100.0)	Period Post-Diagnosis			
		<3 Years	3–5 Years	>5 Years	*p*-Value ^2^
		N (%) 400 (40.7)	N (%) 249 (25.3)	N (%) 334 (34.0)	
**Sex**					0.001
Male	354 (36.0)	124 (29.4)	101 (40.9)	129 (41.1)	
Female	629 (64.0)	298 (70.6)	146 (59.1)	185 (58.9)	
**Age**					0.000
<50	208 (21.2)	112 (26.5)	51 (20.6)	45 (14.3)	
50–65	488 (49.6)	213 (50.5)	127 (51.4)	148 (47.1)	
>65	287 (29.2)	97 (23.0)	69 (27.9)	121 (38.5)	
**Education level**					0.448
<Middle School	156 (15.9)	60 (14.2)	42 (17.0)	54 (17.2)	
High School	351 (35.7)	147 (34.8)	96 (38.9)	108 (34.4)	
>University	476 (48.4)	215 (50.9)	109 (44.1)	152 (48.4)	
**Monthly house income**					0.070
<2,000,000 KRW	275 (28.0)	101 (23.9)	79 (32.0)	95 (30.3)	
2,000,000–4,000,000 KRW	374 (38.0)	166 (39.3)	83 (33.6)	125 (39.8)	
>4,000,000 KRW	334 (34.0)	155 (36.7)	85 (34.4)	94 (29.9)	
**Current employment status**					0.597
Employed	315 (32.0)	135 (32.0)	75 (30.4)	105 (33.4)	
Full-time	65 (19.4)	74 (17.5)	41 (16.6)	50 (15.9)	
Part-time	61 (18.1)	61 (14.5)	34 (13.8)	55 (17.5)	
Self-employed	43 (12.8)	60 (14.2)	34 (13.8)	53 (16.9)	
Unemployed	34 (10.1)	205 (48.6)	124 (50.2)	134 (42.7)	
Others ^1^	133 (39.6)	22 (5.2)	14 (5.7)	22 (7.0)	
**Type of cancer**					0.001
Stomach	153 (15.6)	52 (12.3)	44 (17.8)	57 (18.2)	
Colorectal	207 (21.1)	80 (19.0)	62 (25.1)	65 (20.7)	
Breast	329 (33.5)	166 (39.3)	72 (29.1)	91 (29.0)	
Liver	42 (4.3)	11 (2.6)	10 (4.0)	21 (6.7)	
Lung	57 (5.8)	16 (3.8)	20 (8.1)	21 (6.7)	
Prostate	65 (6.6)	29 (6.9)	16 (6.5)	20 (6.4)	
Gynecological	130 (13.2)	68 (16.1)	23 (9.3)	39 (12.4)	
**Stage of cancer at diagnosis**					0.376
Stage 0	78 (7.9)	28 (2.9)	23 (2.3)	27 (2.8)	
Stage 1	326 (33.2)	135 (13.7)	85 (8.7)	106 (10.8)	
Stage 2	238 (24.2)	98 (10.0)	60 (6.1)	80 (8.1)	
Stage 3	249 (25.3)	110 (11.2)	56 (5.7)	83 (8.4)	
Stage 4	23 (2.3)	10 (1.0)	2 (0.2)	11 (1.1)	
I don’t know/Others	69 (7.1)	19 (1.9)	23 (2.3)	27 (2.8)	
**Smoking status**					0.136
Non-smoker	742 (75.5)	335 (79.4)	175 (70.9)	232 (73.9)	
Past	187 (19.0)	67 (15.9)	55 (22.3)	65 (20.7)	
Current	54 (5.5)	20 (4.7)	17 (6.9)	17 (5.4)	
**Drinking status**					0.003
Non-drinker	267 (27.2)	117 (27.7)	62 (25.1)	88 (28.0)	
Abstained for 1 year	317 (32.2)	164 (38.9)	69 (27.9)	84 (26.8)	
<2 times/week	325 (33.1)	114 (27.0)	94 (38.1)	117 (37.3)	
>2 times/week	74 (7.5)	27 (6.4)	22 (8.9)	25 (8.0)	
**Physical activity frequency**					0.972
5–7 days/week	646 (65.7)	282 (66.8)	163 (66.0)	201 (64.0)	
3–4 days/week	203 (20.7)	85 (20.1)	49 (19.8)	69 (22.0)	
1–2 days/week	87 (8.9)	36 (8.5)	24 (9.7)	27 (8.6)	
None	47 (4.8)	19 (4.5)	11 (4.5)	17(5.44	

^1^ Includes those not actively seeking employment (e.g., housewife, student, etc.). ^2 ^Analyzed by chi-square test.

**Table 2:** Associations of clinical, sociodemographic, and health behavioral factors with anxiety, depression, and quality of life among cancer survivors from the Korean Survey for Cancer Survivorship.

Characteristics	Health Outcomes		
	Anxiety	Depression	Poor Quality of Life
	OR (95% CI)	OR (95% CI)	OR (95% CI)
**Clinical factors**			
**Period post-diagnosis**			
<3 years	1.18 (0.83–1.67)	1.01 (0.75–1.34)	1.55 (1.16–2.08)
3–5 years	1.16 (0.78–1.71)	0.93 (0.66–1.30)	1.17 (0.84–1.63)
>5 years	1.00	1.00	1.00
**Type of cancer**			
Stomach	1.00	1.00	1.00
Colorectal	0.68 (0.39–1.18)	0.83 (0.53–1.30)	1.11 (0.74–1.69)
Liver	1.93 (0.92–4.05)	1.29 (0.65–2.57)	0.92 (0.47–1.82)
Lung	0.96 (0.45–2.06)	1.19 (0.63–2.23)	0.84 (0.46–1.55)
Prostate	1.04 (0.50–2.14)	1.01 (0.54–1.88)	1.04 (0.59–1.86)
Breast	1.78 (1.13–2.81)	2.05 (1.38–3.04)	3.34 (2.25–4.96)
Gynecological	1.27 (0.73–2.23)	1.51 (0.93–2.44)	1.41 (0.88–2.25)
**Socio-demographic factors**			
**Sex**			
Male	1.00	1.00	1.00
Female	1.59 (1.15–2.19)	1.88 (1.43–2.48)	2.72 (2.09–3.55)
**Age**			
<50	3.84 (2.50–5.92)	3.84 (2.62–5.64)	3.02 (2.09–4.37)
50–65	1.76 (1.19–2.61)	2.34 (1.69–3.26)	1.63 (1.22–2.19)
>65	1.00	1.00	1.00
**Monthly house income**			
<2,000,000 KRW	2.05 (1.32–3.20)	2.06 (1.40–3.02)	1.50 (1.02–2.20)
2,000,000–4,000,000 KRW	1.16 (0.79–1.70)	1.09 (0.79–1.50)	1.40 (1.02–1.93)
>4,000,000 KRW	1.00	1.00	1.00
**Current employment status**			
Employed	1.00	1.00	1.00
Self-employed	0.98 (0.60–1.58)	1.10 (0.73–1.65)	0.81 (0.55–1.20)
Unemployed	1.27 (0.91–1.78)	1.38 (1.03–1.84)	1.91 (1.43–2.54)
Others ^1^	1.07 (0.55–2.10)	0.93 (0.52–1.67)	0.99 (0.57–1.71)
**Health behavioral factors**			
**Smoking status**			
Non-smoker	1.00	1.00	1.00
Past	1.52 (0.98–2.34)	1.51 (1.03–2.22)	1.07 (0.74–1.53)
Current	2.46 (1.29–4.70)	1.89 (1.02–3.48)	0.77 (0.42–1.40)
**Drinking status**			
Non-drinker	1.00	1.00	1.00
Abstained for 1 year	1.20 (0.83–1.75)	1.45 (1.04–2.02)	1.34 (0.96–1.86)
<2 times/week	0.89 (0.61–1.31)	1.29 (0.92–1.80)	0.84 (0.61–1.16)
>2 times/week	0.77 (0.41–1.47)	0.87 (0.50–1.51)	0.36 (0.21–0.61)
**Physical activity frequency**			
5–7 days/week	1.00	1.00	1.00
3–4 days/week	1.46 (1.00–2.13)	1.40 (0.99–1.97)	1.43 (1.00–2.03)
1–2 days/week	1.23 (0.71–2.12)	1.57 (0.97–2.55)	1.22 (0.75–1.99)
None	1.04 (0.49–2.21)	0.92 (0.47–1.80)	0.97 (0.51–1.82)

Analyzed by ordinal logistic regression analysis. ^1^ Includes those not actively seeking employment (e.g., retiree, housewife, student, etc.).

**Table 3:** Association of health outcomes among cancer survivors who experienced employment changes ^1^ (N = 332, 46.9%) from the Korean Survey for Cancer Survivorship.

Anxiety	OR (95% CI)	Depression	OR (95% CI)	Quality of Life	OR (95% CI)
None–Minimal	1.00	None–Minimal	1.00	No problems at all	1.00
Mild	1.76 (1.17–2.65)	Mild	2.03 (1.41–2.90)	With some problems	2.89 (2.02–4.13)
Moderate-Severe	3.34 (1.51–7.41)	Moderate-Severe	4.18 (2.28–7.69)	With extreme problems	5.73 (1.76–18.66)

Analyzed by multivariate analysis, adjusted for the effects of age, gender, education level, income level, cancer type, and cancer stage. ^1^ See Figure 3 for changes in employment categories.

## Data Availability

The datasets presented in this article are not readily available because the KSCS is still ongoing. Requests to access the datasets should be directed to the corresponding author.

## Abbreviations

The following abbreviations are used in this manuscript:

KSCS	Korean Survey for Cancer Survivorship
QoL	Quality of life
KNHANES	Korea National Health and Nutrition Examination Survey
EORTC QLQ-C30	EORTC Core Quality of Life questionnaire
EQ-5D-3L	European Quality of Life 5 Dimensions 3 Level Version
PHQ-9	Patient Depression Questionnaire
GAD-7	General Anxiety Disorder
QR	Quick Response
KRW	Korean won
OR	Odds ratio
CI	Confidence interval
CaSUN-K	Cancer Survivors’ Unmet Needs

## Appendix A

**Table A1:** Estimated number of participants of the Korean Survey for Cancer Survivorship by cancer type for follow-up survey in two years.

Cancer Type	Number of Cases	Target Number of Participants	2-Year Survival Rate	Estimated Number of Survivors After 2 Years
Lung	31,616	700	35.5	249
Stomach	29,361	600	70	420
Colorectal	32,751	600	77.9	467
Breast	28,861	600	95.6	574
Liver	18,697	500	90	450
Prostate	15,131	500	49.7	249
Gynecologic	11,143	500	83.8	419
**Total**	**167,560**	**4000**		**2828**

**Table A2:** Survey method by period of the Korean Survey for Cancer Survivorship.

	Survey Year and Target Participants	
	2024–2025 (First Round)	2026–2027 (Second Round, Expected)
Targets of survey	1–5 years after cancer diagnosis: 4000 survivors	2000 survivors for follow-up survey
	5–10 years after cancer diagnosis: 1000 survivors	
Total Target Sample Size	5000	2000
Number of individuals to be contacted	12,500	4000 (from 2024–2025 participants)
